# Aducanumab—Hope or Disappointment for Alzheimer’s Disease

**DOI:** 10.3390/ijms24054367

**Published:** 2023-02-22

**Authors:** Karolina Wojtunik-Kulesza, Monika Rudkowska, Anna Orzeł-Sajdłowska

**Affiliations:** 1 Department of Inorganic Chemistry, Medical University of Lublin, 20-059 Lublin, Poland; 2 Independent Laboratory of Behavioral Studies, Medical University of Lublin, Jaczewskiego 4, 20-090 Lublin, Poland; 3 The Pediatric Department, County Hospital in Lubartów, 21-100 Lubartów, Poland

**Keywords:** Alzheimer’s disease, aducanumab, amyloid, senile plaques, monoclonal antibodies

## Abstract

In June 2021, the world was informed about a new drug for Alzheimer’s disease approved by the FDA. Aducanumab (BIIB037, ADU), being a monoclonal antibody IgG1, is the newest AD treatment. The activity of the drug is targeted towards amyloid β, which is considered one of the main causes of Alzheimer’s disease. Clinical trials have revealed time- and dose-dependent activity towards Aβ reduction, as well as cognition improvement. Biogen, the company responsible for conducting research and introducing the drug to the market, presents the drug as a solution to cognitive impairment, but its limitations, costs, and side effects are controversial. The framework of the paper focuses on the mechanism of aducanumab’s action along with the positive and negative sides of the therapy. The review presents the basis of the amyloid hypothesis that is the cornerstone of therapy, as well as the latest information about aducanumab, its mechanism of action, and the possibility of the use of the drug.

## 1. Introduction

Progress in the world of medicine and pharmacy is visible day by day. The development of modern research and diagnostic methods opens the possibility of quick detection of various diseases, the use of targeted therapy, and a high chance of complete recovery. Unfortunately, in the case of neurological disorders, the development is not as fast as we would like. The problem results from the multi-factorial and complex nature of most of the disorders. One of the more often studied neurological diseases is Alzheimer’s disease (AD) [1].

Alzheimer’s disease has been recognized and characterized for over a century. Despite new diagnostic techniques, the main factor of AD development is still unknown. There are numerous hypotheses that are considered drug targets, but currently, the treatment is still symptomatic and not the causal treatment that we are all waiting for. An additional problem results from the non-specific symptoms of the disease in the early stage of development, which is a time crucial for treatment [2]. The first stage of the disease is characterized by problems with memory (current events and new information). The next step is associated with confusion, disorientation, and behavior changes, as well as depression. In subsequent stages, difficulties speaking, walking, and swallowing occur [3].

Approximately 50 million people around the world suffer from AD, and this number could triple by 2050 if new therapeutic options are not discovered and applied. Long-term studies have revealed numerous risk factors leading to AD development. The most discussed hypotheses are based on amyloid beta (Aβ) formation and changes within the cholinergic system. These two hypotheses are considered the most probable and lead to significant harmful developments in the central nervous system (CNS). Equally important for both the whole organism and, notably, the CNS, is oxidative stress triggered by an imbalance between cellular antioxidants and pro-oxidants that can damage proteins, lipids, and nucleic acid [4,5]. It is known that oxidative stress is especially harmful within the brain due to the organ being rich in unsaturated fatty acids. These are vulnerable to the action of free radicals. Additionally, the brain is rich in metal ions (i.e., iron and zinc), which take part in oxidation reactions. Confirmation of this thesis is the Fritz Haber and Joseph Weiss reaction, being the sum of the Fenton reaction and regeneration of Fe3+ to Fe2+ [6]. Another important phenomenon associated with AD is the alteration of vascular wall function. This can precede amyloid accumulation and AD diagnosis. Pardo-Moreno et al. explained the basis of AD physiopathology in detail [7]. In addition to the aforementioned pro-AD factors, scientists have underlined the importance of tau pathology, in which hyperphosphorylation leads to intraneuronal deposits able to form filamentous aggregates, as well as neuroinflammation, which cause tissue damage and consequently cause neuronal death.

There are numerous environmental factors leading to brain disorders, as well as neurological pathology. Among them are diets low in antioxidants and unsaturated fatty acids, air pollution, smoking and alcohol abuse, and lack of physical and mental activity. All of the aforementioned factors, along with genetic predispositions, induce neurodegeneration development. Current research toward AD drug development is built on the following: Neurotransmitter systems (38%), Aβ pathology (33%), neuroinflammation (17%), tau pathology (10%), and cholesterol metabolism (2%) [8]. In this research, our detailed aims are the inhibition of β- and γ-secretases, potentiation of α-secretase, immunotherapy, tau-directed therapy, antioxidant system development, and regulation of metal level in the organism, i.e., metal chelation [6].

Despite long and detailed studies, only five drugs are available today for AD treatment. Three of these (galantamine, rivastigmine, and donepezil) are based on the inhibition of cholinesterase, the fourth is a memantine, an antagonist of the N-methyl-D-aspartate receptor [7], and the fifth is a new drug—aducanumab—a monoclonal antibody targeted towards Aβ aggregation approved in June 2021 [8]. Pharmacological treatment is currently available, but other drugs are also under research. Among them are γ-secretase inhibitors, β-secretase inhibitors, Aβ immunotherapy, tau aggregation inhibitors, tau phosphorylation inhibitors, and tau immunotherapy. Equally important in the fight against the development of dementia is nonpharmacological treatment, which is a healthy lifestyle (physical exercises, a Mediterranean diet, and good sleeping habits) [9].

Considering the multifactorial characteristics of AD, an interesting solution is the concept of precision medicine. This relatively new conception is based on the theory of an individual approach to each patient rather than a general approach to the disease as has been the case so far [10]. The approach is characterized by an individual’s genetic makeup and custom tailoring therapy, which will be directed toward the specific cause of the disease, which will be diagnosed and confirmed by the available methods. The National Institute of Health (NIH) described the approach as evolutionary, which allows us to eliminate the ‘one size fits all’ criteria of diseases. In the case of AD, genetic polymorphisms are extremely important for the precision medicine approach. It is known that the disorder is strictly associated with apolipoprotein E, which is considered the main genetic factor in late-onset Alzheimer’s [11]. Thus, in this case, precision medicine can be targeted toward the APOE genotype as an AD single-factor investigation [12].

Another important gene associated with AD development is the methylenetetrahydrofolate reductase (MTHFR) protein-encoding MTHRD gene. Polymorphism of the gene can impact AD via the MTHFR catalytic function, which is a rate-limiting step in the transformation of homocysteine to methionine. Homocysteine plays a significant role in cognitive decline and inflammation, which elevate AD risk [13]. The significant elements are B vitamin folate and cobalamin, which play the role of cofactors. The study results revealed a positive influence of B-vitamin supplementation, which caused a reduced homocysteine concentration. The supplementation can be considered dementia prevention for the elderly with high homocysteine levels [14]. Considering the precise medicine, individuals with MTHR polymorphisms can be recommended for therapy. Nevertheless, precise medicine identification of latent pathophysiological processes in AD is based on brain imaging technologies. Recently, these technologies have developed significantly, which provides great opportunities for the development of this type of medicine.

It is worth mentioning the significant impact of calcium dysregulation on the pathogenesis of AD. It is known that presenilin mutations can impact changes in intracellular calcium homeostasis. There are a few possible mechanisms: Enhancing the function of IP3R, PyR, and TRPC or affecting CCE pathways, inhibiting the function of presenilin-mediated calcium release, and encouraging the lysosomal calcium release [15,16,17]. In vivo studies based on transgenic animal models revealed that the production and aggregation of Aβ and the increase in the phosphorylated tau protein can be induced by the administration of Ca^2+^. Additionally, an imbalance of the ion can cause metabolism dysregulation resulting in neuroinflammation, neurotoxicity, or autophagy [17,18].

The review paper is focused on one of the most intensively studied AD hypotheses, namely, the amyloid hypothesis, which is the basis of the drug activity of aducanumab. The FDA’s acceptance of this drug caused much controversy in the scientific community. There are many facts for and against taking this drug, which will be presented in the submitted paper. The aim of the review paper is the presentation of the positive and negative sides of aducanumab. There are available study results that revealed the activity of the drug against β-amyloid, but the drug is also presented as bringing more negatives than positives. This publication may help to address this new drug.

## 2. Search Strategy

**Figure 1:**
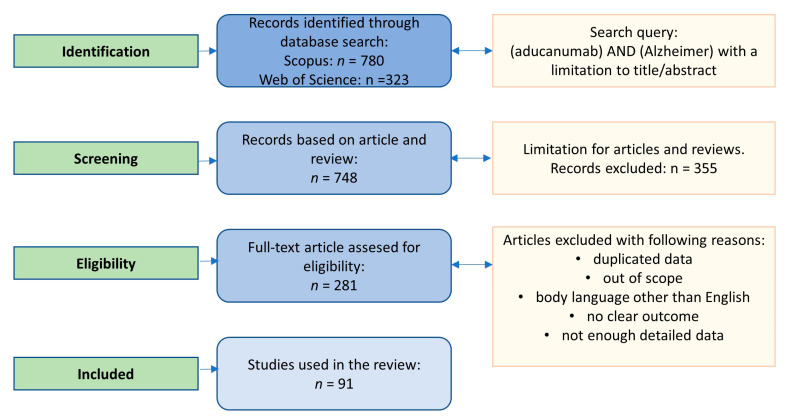
Search strategy for the review paper.

The search strategy for the review involved Scopus and Web of Science databases. The search was based on the terms “aducanumab” in combination with “Alzheimer”. There was no time frame. The articles were verified as presented in Figure 1.

## 3. Introduction to Amyloid Beta

One of the most often discussed and studied pro-neurodegenerative factors of AD is amyloid beta (Aβ). It is known that early-onset AD (5–6% of all cases) is closely related to mutations in APP (amyloid precursor protein), apolipoprotein E (ApoE-ε4), and presenilin (PSEN1 and PSEN-2) [19]. Aβ can be found not only in CNS but also in the plasma, bone, and other organs [20]. The development of amyloid structures is considered one of the most probable and fundamental pillars of AD and is the basis of the amyloid cascade hypothesis. This postulates that the development of AD results from the formation and aggregation of deposits of amyloid peptides. These structures lead to the dysfunction of neurons, which, in turn, has a negative impact on learning and memory [19]. The first link in the ‘amyloid puzzle’ is the amyloid precursor protein (APP)—a transmembrane glycoprotein. There are three isomers of APP, namely, 697, 751, and 770. According to study results, APP695 is expressed in the neurons, glial cells, and other peripheral cells, while APP751 is expressed in glial cells and other peripheral cells, and APP770 is expressed in the vascular endothelial cells [20].

Researchers have indicated APP695 to be of the greatest importance in Aβ formation. It is composed of an extracellular domain (Aβ domain) and a cytoplasmic region, which interacts with various proteins [21]. There is little information about the physiological significance of the protein, but its influence on amyloid accumulation is due to its creation of proteolytic cleavages via an amyloidogenic or a non-amyloidogenic pathway, leading to the creation of protein fragments made up of various numbers of amino acids. The processes are strictly connected to enzymes (secretases) responsible for the cleavage of the protein. In a normal, physiological situation, APP acts upon α-secretase, leading to the production of a soluble fragment (sAPPα) which remains in the extracellular space as an 83-amino acid fragment (a CTFα) and is anchored in the plasma membrane. The last fragment can be acted upon by γ-secretase, leading to extracellular P3 pieces and an APP intracellular domain (AICD). What is interesting is that sAPPα can improve synaptic plasticity, learning, and memory, in addition to counteracting metabolic stress [22].

**Figure 2:**
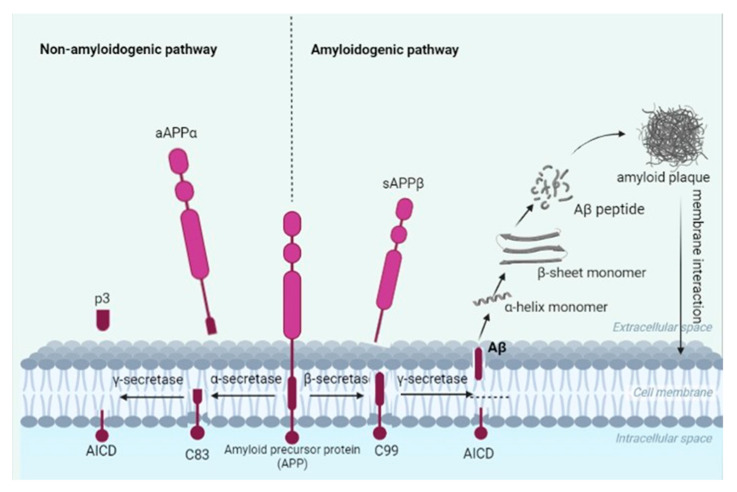
Non-amyloidogenic and amyloidogenic pathways of amyloid precursor protein (APP) cleavage. The non-amyloidogenic pathway leads to the formation of an 83-amino acid fragment (a CTFα), which, when treated with γ-secretase, forms a two-fragment p3 and AICD, being an APP intracellular domain. These forms are not toxic and are not able to form senile plaques. The amyloidogenic pathway, however, is a toxic form of APP cleavage and leads to the formation of a soluble aAPPβ fragment and a C-terminal part composed of 99 amino acids. The latter, under γ-secretase activity, creates Aβ, which is able to aggregate towards senile plaques.

More important for human well-being is the amyloidogenic pathway of APP cleavage. In this case, the first stage is based on APP cleavage by membrane-bound aspartyl protease (BACE1, β-secretase), leading to the creation of soluble aAPPβ fragments and a C-terminal part composed of 99 amino acids (CTFβ or C99). The last one is cut by γ-secretase into Aβ peptides primarily consisting of 39–42 amino acids. The Aβ42 peptide in this case is more toxic and more prone to aggregation [19,21]. The pathways are presented in Figure 2. The neuropathological situation leads to the creation of the aforementioned amyloid beta, which has an influence on the neuron condition. In accordance with Bressler et al. [23], the C99 fragment can impact neuronal death by apoptosis via the indirect induction of gene expression. A stream of research on the amyloidogenic pathway assumes that APP is re-internalized into endosomes containing β- and γ-secretases, so that once synthesized, Aβ peptides can be exported to the extracellular space or degraded in lysosomes. In the majority of cases, however, Aβ is released into the intracellular space, primarily affecting the axon, but with a minor effect on the dendrites [19,20,24].

Amyloidogenesis can be described as a form of nucleation-dependent polymerization, and time-wise consists of an energetically unfavorable lag phase, an elongation phase, and a plateau phase when the fibril extension ends [25]. Regarding the structure of Aβ, Aβ40 and Aβ42 monomers are random coil or α-helix structures, while Aβ fibers, under specific environmental conditions, transform from their natural structure into β-sheets [26]. As a result, the structures evolve oligomers, protofibrils, and fibrils, which, along with plaques, are able to accumulate around neuronal cells and lead to the impairment of membrane functions and neuronal transition [27]. Aβ40 can exist as dimers, trimers, and tetramers, but they are incapable of creating hexamers, whereas Aβ42 is able to create pentamers and hexamers of a planar hexagonal structure, as well as two subnuclear hexamers, which are combined into a stacked sub-nuclear dimer [28].

Numerous components of the neurovascular unit such as neurons, perivascular astrocytes, microglia, pericytes, endothelial cells, and the basement membrane are exposed to the toxic effects of amyloids [29]. Senile plaques were under consideration for many years as structures leading to vascular damage and neuronal loss [22]. However, oligomers of Aβ can also impair cognitive functions, and soluble oligomers can create various structures such as dimers, trimers, and tetramers. Study results reveal that neurodegeneration can be caused not by the largest aggregates, but by oligomer structures between 3 and 10 nm [30].

The toxicity and impact of Aβ on the organism depend on its level within the brain. At physiological levels, the soluble amyloid is used for the regulation of synaptic plasticity, as well as for neuronal survival. This role changes as their levels in the brain change, namely, too low a level can lead to reduced synaptic activity presynaptically, whereas intermediate levels have opposite effects. The most destructive is a too-high level of amyloid, which leads to the accumulation of the peptide in the intra and extracellular space, forming toxic intermediates and Aβ oligomers [31,32]. Dimers of the amyloid accumulate in lipid rafts, which facilitates the aggregation and formation of senile plaques.

**Figure 3:**
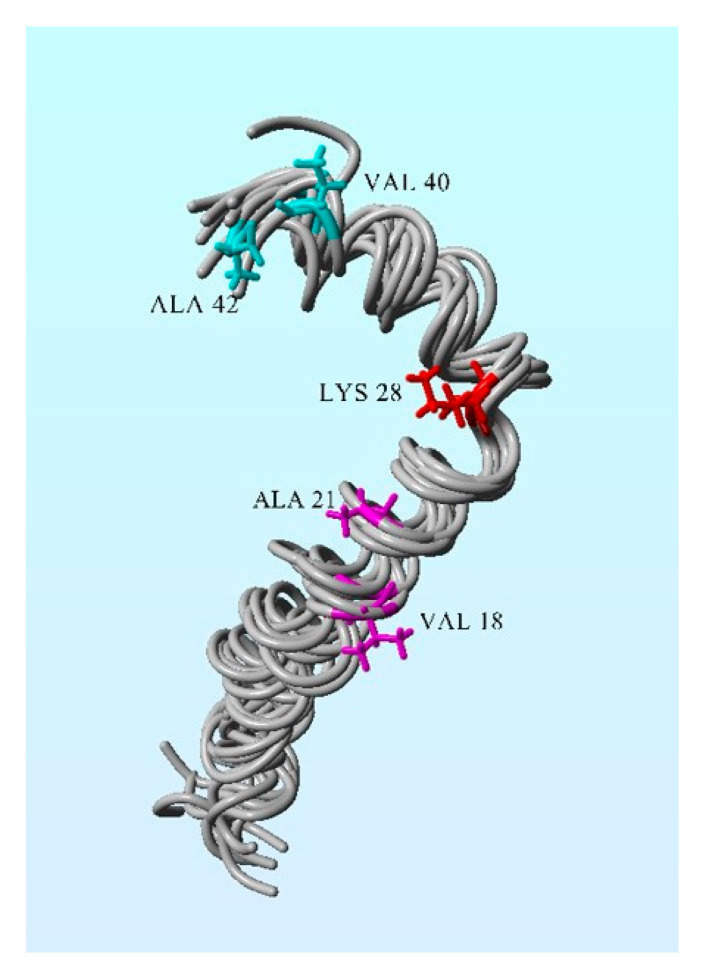
Solution structure of the Alzheimer’s disease Aβ (1–42). The following hydrophobic groups of amino acid residues are engaged in Aβ toxicity: Ala21–Val18, Val40-Ala42, and Lys28. Figure preparation: PDB ID: 1IYT, Yasara 11.2.15 package (Yasara Bioscience, Graz, Austria).

The toxicity of amyloid is explained by four possible methods of Aβ action [20,33]:

- Binding by hydrophobic interaction (engaging three hydrophobic groups: Val18-Ala21, Lys28, and Val40-Ala42), which can affect cell viability (Figure 3).
- Encouraging the production of more amyloid fiber by releasing two short fragments able to replicate.
- Enhancing amyloid production by mitochondrial involvement, which can lead to dysfunction of ER and mitochondria.
- Forming new Aβ oligomers, which can generate higher-toxicity effects.

The accumulation of amyloid β is presented as a factor responsible for the formation of senile plaques showing a toxic effect on the brain. Still, there is some evidence suggesting that senile plaques are associated with cerebral microhemorrhages (known also as cerebral microbleeds) [34]. Cerebral microhemorrhaging is presented as an important cause of neurodegeneration leading to AD. It is assumed that brain hemorrhage is strongly associated with the cerebrovascular deposition of Aβ [35]. Numerous studies have revealed explicit associations between heme-rich deposits (HRD) and Aβ [36,37,38]. In the studies described by Casey, for example, 14 out of 20 individuals affected with AD revealed Aβ deposits localized with HRDs [39]. Confirmation of this theory can be found in study results that revealed that vascular amyloid is a pivotal step for cerebral amyloid angiopathy (CAA)-related hemorrhage, and, hence, reducing and preventing brain hemorrhage can be obtained by reducing amyloid burdens [35]. However, it is puzzling that only 23% of all studied AD patients exhibit brain hemorrhage, whereas 90% of all patients with AD include CAA in the brain (based on autopsy) [40].

Many studies explicitly indicate the multifactorial character of AD, including both the amyloid and cholinesterase hypotheses [41,42,43]. Their coexistence can be confirmed by the fact that senile plaques contain molecules of the synaptic form of human AChE (hAChE-S) [25]. This coexistence can also be confirmed by the presence of hAChE-S, which is associated with AD plaques and tangles, as well as in vivo and ex vivo studies, which revealed earlier development of the disorder in double transgenic mice (both Aβ accumulation and cholinergic impairment) than in single transgenic mice [44,45].

Considering the long-lasting studies around the world, there are numerous pieces of evidence supporting the amyloid hypothesis [46]:

- Aβ is always a feature of AD, but NFTs are not.
- Amyloids under elevated concentration are a neurotoxin in tissue culture.
- Fibrillar amyloid beta can induce mitogen-activated protein kinase, leading to tau phosphorylation and the formation of neurofibrillary tangles.
- An increase in the level of Aβ is associated with mutations in the APP, PS1, and PS2 genes.
- In vivo studies based on transgenic mice revealed a correlation between the Aβ concentration and amyloid plaques [47].
- Treatment of Aβ leads to NFT clearance in the early stages [48].

Nevertheless, more and more studies report the lack of a correlation between cognitive impairment and senile plaques [19]:

- Aβ oligomer levels per plaque are much lower than in AD brains, which indicates that plaques can sequester oligomers in a non-diffusible, less neurotoxic state [49].
- The physiological concentration of Aβ does not play a neurotoxic role in the organism [50].
- Cell death is not caused by the presence of only amyloid plaques, whereas the presence of tau is always associated with neurodegeneration [32].
- Moreover:
- Clinical studies did not always indicate a correlation between the presence of plaques and AD [51].
- Lowering amyloid levels through immunotherapy against the amyloid caused harm to the recipients, including neuroinflammation [52].
- Reduction of Aβ levels did not impact behavioral changes (water-maze and Y-maze tests with transgenic animals) [53].
- Individual differences exist in the ability of inflammatory cells to effectively clear senile plaques in the brain [51].
- Individual variations in brain plasticity and the ability to restore brain function after an injury have been noted [54].
- The neurotoxic properties of amyloid oligomers precede the less neurotoxic senile plaques and could very likely be the main cause of cognitive impairment [55].

## 4. Monoclonal Antibodies in AD Therapy

Therapies based on monoclonal antibodies (mAbs) gained popularity in 1975 when Köhler and Milstein developed methods for their isolation from hybridoma cells [56,57]. The scientists did not patent their production method, which facilitated the use of the technology by other researchers and the industry, hence facilitating therapy development. The mAbs are produced by B cells and, specifically, target antigens [58]. The FDA has accepted more and more therapies based on this solution. The first was muromonab (OKT3), an anti-CD3 monoclonal antibody used to counter organ transplant rejection [39].

There are four main categories of monoclonal antibodies:

1. **Murine antibodies**: Procured entirely from mouse proteins, they are recognized as allogeneic proteins, hence leading to polyclonal human anti-mouse antibody (HAMA) reactions, usually 2–3 weeks after their initial infusion [56]. Currently, the antibodies are not used in neurology.
2. **Chimeric antibodies**: The characteristic feature of the antibodies is the fact that they contain only 34% mouse proteins in variable regions of the antibody. This has an impact on the lower incidence of the HAMA reaction in comparison to murine mAbs. Additional advantages are the longer half-life and increased affinity for the antigen, which creates better pharmacodynamic and pharmacokinetic profiles. In neurology, only rituximab and infliximab are used [59,60].
3. **Humanized antibodies**: The antibodies are 90% human and 10% mouse protein. In this case, they are less immunogenic and acquire biological functions, along with retaining the specificity and binding affinity of the ‘parental’ murine mAbs [61]. These antibodies are commonly used in neurological indications.
4. **Fully Human Monoclonal Antibodies**: New technologies and transgenic mice allowed the production of 100% human mAbs. The antibodies are characterized by the complete removal of murine components. This has led to fewer immunogenic reactions, as well as better pharmacokinetic profiles. Today, fully human mAbs are used in migraine and multiple sclerosis therapy (erenumab and ofatumumab, respectively) [58].

Monoclonal antibodies can act via several mechanisms: Direct and indirect or immune-mediated actions. Direct mechanisms are based on blocking ligand–receptor interactions via binding to a soluble ligand or receptor or a cell-bound ligand or receptor, leading to the inhibition of downstream signaling events, or agonism through binding to a receptor by mimicking its natural ligand, leading to the activation of signaling pathways. Indirect mechanisms are immune-mediated as they involve the activation of certain types of immune cells and molecules to kill target cells [56,60,62].

It seems that effective AD treatment should be based on the amyloid hypothesis. Herein, one possible way to reduce the amyloid is through the breaking of peptides. This was the approach used in the development of the first anti-amyloid breaker peptides, which targeted the segment 18V-E22 of Aβ(1–42) so as to reduce Aβ [63]. The solution was used in studies for the reduction of Aβ. The most promising solution turned out to be immunotherapy based on monoclonal antibodies. 

Immunotherapy based on monoclonal studies has proven to be a promising solution due to its high affinity and specificity. Unfortunately, the approach requires high financial outlay [64].

In such research, the first stage of studies should answer the question of whether amyloid is truly a critical point in the development of the disease. A few therapies based on Aβ immunotherapy failed, and this outcome explicitly indicates that the strategy must be modified. Considering the fact that amyloidogenic proteins (AP) such as p53, amylin, and adrenomedullin have a significant impact on AD development, therapy based on singular AP is insufficient [65]. Ho et al. [65] proposed a more practical solution to the problem. They recommended investigating activin, with this being TGF-β family member signaling through the type II and -I receptors. Activin is known to be involved in protein aggregation in AD, thus the down-regulation of activin is desirable.

To date, a few monoclonal antibodies were tested for anti-neurodegeneration, including anti-Aβ. Table 1 presents the basic characteristics of the molecules tested for AD.

## 5. Aducanumab–Positive and Negative Sides of Therapy

Alzheimer’s disease is an irreversible CNS disorder. Up to June 2021, medicine had in its arsenal only four drugs based on AChE inhibitors and an NMDA agonist. The situation changed when the FDA approved a new drug based on the amyloid hypothesis. 

**Figure 4:**
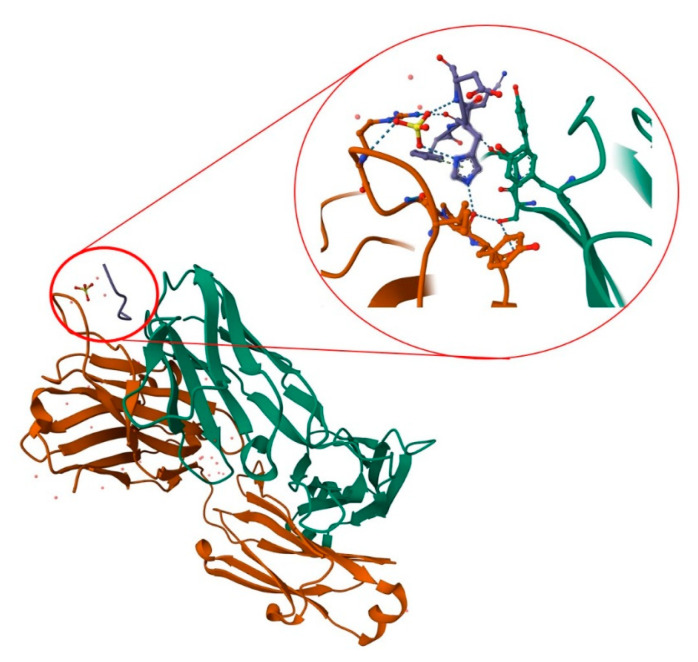
Structure of AduFab with bound Aβ (1–11) peptide. The figure shows Fab light chain in green, Fab heavy chain in brown, and amyloid β in blue, along with hydrogen interactions between AduFab and amyloid. Prepared with the use of Protein Data Bank.

Aducanumab is a recombinant, human immunoglobulin G1 monoclonal antibody targeting soluble amyloid beta and insoluble fibrils [76]. The antibody was derived from a blood lymphocyte library from elderly people without cognitive impairment or with unusually slow cognitive decline. In 2018, Arndt et al. presented the structure of the aducanumab amyloid beta complex [77]. Further research provided a structural rationale for the low affinity of the molecule for non-pathogenic monomers (Figure 4). In later work, in silico studies allowed the analysis of the structure of the molecule, along with its interaction with the amyloid (residues 1–11). It is now known that aducanumab is able to bind Aβ residues 3–7 in an external conformation. Further work has led to the crystallization of Fab from aducanumab (AduFab). The most important amyloid residues interacting with AduFab are Phe4 and His6, along with Glu3, while the main-chain carbonyl of Arg5 makes additional contributions to the binding interaction.

A characteristic feature of aducanumab is its ability to bind oligomeric and fibrillar states of amyloid rather than monomers. The monoclonal antibody provides specific amino acid interactions, which allow for more shallow and compact binding in comparison to other monoclonal antibodies [78].

In vivo research is an obligatory stage of research on a new drug. Similar to other studied substances, the activity of aducanumab was analyzed with the use of mice models (22-month-old mice genetically modified to overexpress APP). The studies were performed in acute and chronic (6 months) models. Changes in the brain were observed with the use of fluorescent microscopy to tag Aβ plaques. Similarly, before and after treatment, the inositol triphosphate receptor, NMDA receptor, ryanodine receptor, and visinin-like-protein activity were observed. Detailed analysis of the obtained results revealed that acute treatment caused a greater decrease in amyloid plaques than the placebo. A reduction was observed to 48% of the total number of Aβ plaques, whereas the control group revealed only a 14% reduction. However, chronic treatment did not bring a significant reduction of amyloid plaques [16,78].

The specificity of aducanumab is the fact that it is the only drug based on Aβ. The amyloid hypothesis has many contradictions and therefore the drug is controversial. The drug’s history had its start in 2016 when Biogen reported data from the phase 1b PRIME trial, which revealed a reduction of the amyloid burden in the brain (10 mg/kg) (details: Section 5.1). Additionally, a positive influence on cognition was observed [79]. Aducanumab was subjected to subsequent trials (EMERGE and ENGAGE), and while ENGAGE did not reveal a positive effect in comparison with the placebo, EMERGE revealed amyloid reduction and was supported by an ad hoc analysis (details: Section 5.2) [76]. The study results revealed dose-dependent and time-dependent amyloid reduction. It is important to mention that EMERGE was based on a small group of patients who were in treatment for at least 14 months. Despite the controversy surrounding the conducted clinical trials, the FDA approved aducanumab (ADUHELM^®^, 100 mg/mL solution) for the treatment of AD [80].

### 5.1. Phase 1b: PRIME

It is commonly known that the approval of a new medicinal substance and drugs is preceded by many years of research based on in vitro, in silico, and in vivo tests, which end with clinical tests. Significant in vivo studies of aducanumab were presented by Sevigny et al. in 2016 [66]. To date (January 2023), the paper has been cited 1591 times, which underlines the importance of the presented study results. The researchers presented the interim results from a double-blind, placebo-controlled phase 1b trial. The aim of the studies was to evaluate the safety, tolerability, pharmacokinetics, and pharmacodynamics of aducanumab. The PRIME phase was based on 165 patients with diagnosed prodromal or mild Alzheimer’s disease and confirmed by positive emission tomography (PET) scans of amyloid beta in the brain. The outcome of the study explicitly indicated a positive impact of aducanumab on Aβ reduction in a dose- and time-dependent fashion. The trial, which lasted 54 weeks, brought about a significant decrease in the PET standard uptake value ratio (SUVR) in the 3, 6, and 10 mg/kg dose groups, in comparison to the baseline, whereas the placebo group was not significant. Equally interesting and important is that the trial established that aducanumab can penetrate the brain to a sufficient extent to allow the accumulation of Aβ plaques. What is more, aducanumab was found to be able to clear plaques of all sizes, which suggests that the substance is able to prevent the formation of new plaques [66].

Nevertheless, it is worth mentioning the limitation of the studies. The PRIME phase 1b was based on a small sample size, was conducted in the USA only, had a staggered parallel-group design, and indicated possible partial unblinding due to ARIA-E (vasogenic edema). Moreover, ARIA-E was observed in 1 (3), 2 (6%), 11 (37%), and 13 (41%) participants who were treated with 1, 3, 6, and 10 mg/kg, respectively. The trial was also continued with more than half (56%) of all participants displaying the aforementioned side effect. However, referring to these limitations, the researchers underlined the results of their post hoc analysis, which indicated no apparent differences in treatment effect when comparing patients with and without ARIA-E. Other side effects of the therapy were headaches, urinary tract infections, and upper respiratory tract infections [66].

### 5.2. Phase 3: ENGAGE and EMERGE

Phase 3 studies were conducted with 1600 amyloid-positive participants with early AD in each trial. The trials involved adults and older adults (50 years to 85 years) who met a number of criteria. Among these were the following: Objective evidence of cognitive impairment at screening, a Mini-Mental State Examination (MMSE) score between 24 and 30, a positive amyloid PET scan, and a Clinical Dementia Rating (CDR)–Global score of 0.5, as well as having a reliable informant or caregiver. In the case of patients who were treated with AD drugs, doses had to be stable for at least 8 weeks prior to their first screening visit [81]. The most important exclusion criteria were the following: Clinically significant unstable psychiatric illness in the past 6 months, impaired renal or liver function, taking blood thinners, except aspirin, at a prophylactic dose or less, brain hemorrhage, bleeding disorder, and cerebrovascular abnormalities, any condition other than AD conditions that can influence cognitive impairment, and having a stroke or Transient Ischemic Attack or unexplained loss of consciousness in the past 1 year. Dosage differed from the recommendation for the trials, namely, EMERGE participants were treated with higher doses for longer periods.

The study results revealed no drug–placebo difference for primary and secondary clinical outcomes in the final dataset of the ENGAGE trial. Moreover, differences were observed between the ENGAGE and EMERGE trials; notably, ENGAGE did not have positive results. The trial did not reveal a benefit in comparison to the placebo. In contrast, the EMERGE trial revealed a 22% decreased rate of cognition impairment in the group of patients treated with high-dose of aducanumab (10 mg/kg) [78]. The FDA then performed post hoc analysis, which revealed a decrease in the amyloid burden: Low-dose aducanumab = 0.179 reduction in mean SUVR; high-dose aducanumab: 0.278 reduction in mean SUVR [*n* = 109], placebo = no change [*n* = 93] [82]. In both trials, adverse events were observed. Herein, amyloid-related imaging abnormalities occurred in 34% of the test population in the EMERGE group and 35.5% in the ENGAGE group. Both ENGAGE and EMERGE trials also revealed ARIAs, which occurred within eight doses (7 months of initiation). It should be underlined that almost all ARIA-E cases were resolved within 3 months (69%) and 4 months (83%). Patients who suffered from ARIAs also revealed other symptoms such as headache (47%), confusion (15%), dizziness (11%), and nausea (8%). All patients were treated with the highest dose of 10 mg/kg [83].

Clinical trials of aducanumab in patients with Alzheimer’s diseases listed on Clinicaltrials.gov (accessed on 16 January 2023) are presented in Table 2.

An interesting fact is that aducanumab can impact calcium homeostasis, of which dysregulation is one of the possible pro-AD factors. Based on in vivo studies performed on 2756 transgenic mice, aducanumab caused restoration of calcium homeostasis. Treatment of cognitive impairment resulting from the mitigation of overload of calcium was observed [16,84,85]. Aducanumab administration in 22-month-old mice did not clear existing plaques whereas calcium overload was ameliorated over time. Analysis of the obtained results suggests that expression of the intracellular store channel was reduced in Tg2576 mice treated with the control antibody and restored with aducanumab immunotherapy, which suggests that intracellular calcium stores may contribute to calcium dyshomeostasis [16].

It is known that the effective action of drugs is possible after reaching the appropriate concentration in the treated organ, which, in the case of the brain, is very difficult due to the blood–brain barrier. Study results revealed that the maximal effectiveness of aducanumab was observed around the fifth month of the therapy, which results from the establishment of the appropriate concentration of the substance that will be able to induce the destruction of amyloid aggregates. Pharmacodynamic analysis revealed that aducanumab binds fibrils and targets them for microglial-mediated removal, interrupting the bridge between neuroprotective amyloid monomers and neurotoxic oligomers [78,86].

## 6. Negative Aspects of Aducanumab Approvement

The drug is controversial for several reasons. It is commonly known that AD is a multifactorial disorder and there are a few hypotheses that lead to the development of the disease. Hence, therapy based on one potential factor is not satisfactory. In the anti-aducanumab camp, one of the most commonly quoted reasons for their position is that a test based on patients with mild biomarker-proven AD cannot reliably confirm the activity of the substance. Furthermore, the FDA did not give guidance as to which patients can be treated with the drug [79]. In addition, the ENGAGE phase did not reveal aducanumab to have spectacular activity. Indeed, the substance only revealed a comparable result to the placebo [87]. Moreover, with regard to side effects, there are study results that revealed amyloid-related imaging abnormalities (ARIAs), which can be due to vasogenic edema of the brain leading to microbleeds. In accordance with Haeberlein et al. (2019) [88], over 40% of patients taking the drug have experienced ARIAs, while 7.5% were symptomatic [66]. Considering therapy, ARIAs must be monitored. Patients displaying possible amyloid angiopathy associated with microbleeds should be excluded from therapy.

The obligatory exclusion aspect of aducanumab treatment is the abnormal amyloid present in the brain. In accordance with the available data, in the case of approximately 20–40% of all patients suffering from early AD, abnormal amyloid is not observed. Hence, in the case of these people, therapy based on aducanumab is ineffective [89]. Overall, it must be said that additional research (i.e., amyloid PET and CSF) is needed to ensure that the new therapy would be effective. This solution generates additional costs. 

Another negative aspect of the therapy is linked to the aforementioned costs. The drug is administered in the form of monthly infusions, the costs of which are estimated to be approximately $56,000 per year. Noteworthily, because of low sales, the costs have been reduced (December 2021) to $28,000 per year, but the price is still too high for most people [80]. Finally, to date, the FDA has only approved drugs that included detailed studies and revealed high activity, along with in-depth statistical analysis. In the case of aducanumab, the FDA approved the substance without two good-quality studies, and only on the basis of the high-dose arm in study 302 upon post hoc analysis. What is more, the FDA approved the drug despite the negative opinion of the organization’s biostatistics reviewers [90].

Taking into consideration the side effects, high costs, and limited actual effect, the therapy does not have a good reputation. The turning point may be the moment of presenting the research results from a phase IV (post-marketing) trial, which is intended to determine the usefulness of the therapy. A significant aspect of the drug’s approval is the limitation of performed studies. One of the most important is the fact that aducanumab was tested in patients with early and mild AD, whereas the advanced form of the disorder can be a critical point of approval. An equally important limitation of the treatment is the lack of guidance on which AD patients can be treated, and the decision should be made by the Center for Medicare & Medicaid Services [79]. A significant limitation can also be the origin of patients who took part in clinical studies. In accordance with the available data, the patients did not vary in terms of origin, which could affect the differences in the activity of the tested compound [90].

## 7. Conclusions

The amyloid hypothesis has been the subject of study for many years, but the obtained results have not explicitly indicated the factor as dominant in AD development. While aducanumab, a drug based on the theory, has revealed satisfactory activity, the new therapy can be used only by a limited group of patients and is insufficient in countering neurodegeneration. An additional aspect is the multifactorial character of AD, which is underlined in most scientific papers and presented as the only effective way of advancing AD treatment. Unfortunately, aducanumab can only act in one direction and is without activity towards the equally important cholinergic pathway or other pro-neurodegenerative factors such as oxidative stress. An important direction of studies based on aducanumab is precision medicine. It is known that the drug is active towards amyloid β and calcium dyshomeostasis, thus the development of the therapy in these two directions is extremely important. The development of precision medicine could be the solution to the problem of AD treatment and the controversial aspects of aducanumab. Currently, it is hard to explicitly evaluate the decision of the FDA. The rightness of the decision can be assessed only after a few years following the introduction of therapy, but what if this path turns out to be wrong?

## Figures and Tables

**Table 1:** Recent monoclonal antibodies studied against Alzheimer’s disease.

Name	Type	Action	Stage of Development	Advancement of Alzheimer’s Disease	References
Aducanumab	Fully human IgG1	Against Aβ aggregation	Accepted by FDA	Prodromal to mild	[66,67]
Donanemab	Humanized IgG1	Binding aggregated Aβ forms	In phase III	Mild	[68,69]
Gantenerumab	Fully human IgG1	Binding aggregated Aβ forms	In two phase III Trials, The company stopped all trials in order to prepare a new Gantenerumab formula.	Prodromal to mild	[70,71]
Gosuranemab	Humanized IgG4	Targeting abnormal forms of tau protein or soluble oligomers	Negative results in phase II	Prodromal to mild	[72,73]
Semorinemab	Humanized IgG4	Targeting all isoforms of tau protein	A phase 3 decision is pending	Prodromal to mild	[72,74]
Tilavonemab	Humanized IgG4	Targeting abnormal extracellular forms of tau protein	Trial development was stopped after phase II (2020)	Prodromal to mild	[72,75]

**Table 2:** Data obtained during aducanumab clinical trials.

Study Name/Identification	Number Enrolled	Key Inclusion Criteria	Level of Evidence Statement
Single ascending dose study of BIIB037 in participants with AD	53	Clinically confirmed AD, age: 55–85 years old, others: Good health, reliable informant or caregiver	Single dose of aducanumab (up to 30 mg/kg) was safe and tolerable
PRIME (Multiple dose study of aducanumab)	197	Prodromal or mild AD, Age: 50–90 years old; others: Good health, reliable informant or caregiver	Decreasing amyloid value studied with the use of PET SUVR at 1 year vs. placebo (dosage: 3–10 mg/kg)
ENGAGE (Phase 3 Study)	1647	MCI due to AD or mild AD; Age: 50–85 years old; MMSE 24–30; others: Positive amyloid PET scan, stable doses of drugs treating AD symptoms, reliable informant or caregiver	Aducanumab (3–10 mg/kg) did not significantly affect mean change in CDR-SB scores vs. placebo over 78 weeks whereas the same doses caused decrease in amyloid PET SUVR at 78 weeks vs. placebo
EMERGE (Phase 3 study)	1638	MCI due to AD or mild AD; Age: 50–85 years old; MMSE 24–30; others: Positive amyloid PET scan, stable doses of drugs treating AD symptoms, reliable informant or caregiver	Aducanumab at a dose of 10 mg/kg results in less worsening of the CDR-SB vs. placebo at 78 weeks; degree less than a clinically relevant change; doses of 3–10 mg/kg caused decrease in amyloid PET SUVR at 78 weeks vs. placebo
EVOLVE	52	MCI due to AD or mild AD; Age: 50–85 years old; MMSE 24–30 others: Positive amyloid PET scan	NA
PROPEL (Single and multiple ascending dose study in Japanese participants with AD)	21	Clinical diagnosis of mild-moderate AD; age: 55–85 years old; others: Good health, reliable informant or caregiver	NA

## Data Availability

Not applicable.
